# The impact of level of documentation on the accessibility and affordability of new drugs in Norway

**DOI:** 10.3389/fphar.2024.1338541

**Published:** 2024-02-14

**Authors:** Gro Live Fagereng, Anne Marit Morvik, Sara Reinvik Ulimoen, Anne Marthe Ringerud, Iselin Dahlen Syversen, Erik Sagdahl

**Affiliations:** ^1^ The Pharmaceutical Division, The Norwegian Hospital Procurement Trust, Vadsø, Norway; ^2^ Institute for Cancer Research, Division of Cancer Medicine, Oslo University Hospital, Oslo, Norway; ^3^ Norwegian Medical Products Agency, Oslo, Norway; ^4^ South-Eastern Norway Regional Health Authority, Hamar, Norway; ^5^ Department of Medical Research, Bærum Hospital, Vestre Viken Hospital Trust, Drammen, Norway; ^6^ Department of Pharmacy, The Arctic University of Norway, Tromsø, Norway

**Keywords:** drugs, net-prices, reimbursement, managed-entry agreement, oncology, medicinal product, European Medicines Agency, Health Technology Assessment

## Abstract

**Introduction:** Over the preceding decade, an increasing number of drugs have been approved by the European Medicines Agency (EMA) with limited knowledge of their relative efficacy. This is due to the utilization of non-randomized, single-arm studies, surrogate endpoints, and shorter follow-up time. The impact of this trend on the accessibility and affordability of newly approved drugs in Europe remains uncertain. The primary objective of this study is to provide insights into the issues of accessibility and affordability of new drugs in the Norwegian healthcare system.

**Method:** The presented study entails an analysis of all reimbursement decisions for hospital drugs in Norway spanning 2021–2022. The included drugs were approved by the EMA between 2014 and 2022, with the majority (91%) receiving approval between 2018 and 2022. The drugs were categorized based on the level of documentation of relative efficacy. Approval rates and costs (confidential net-prices) were compared.

**Results:** A total of 35% (70/199) of the reimbursement decisions were characterized by limited certainty regarding relative efficacy and as a consequence the Norwegian Health Technology Assessment (HTA) body did not present an incremental cost-effectiveness ratio (ICER) in the HTA report. Within this category, a lower percentage of drugs (47%) gained reimbursement approval compared to those with a higher certainty level, which were presented with an ICER (58%). On average, drugs with an established relative efficacy were accepted with a 4.4-fold higher cost (confidential net-prices). These trends persisted when specifically examining oncology drugs.

**Conclusion:** Our study underscores that a substantial number of recently introduced drugs receive reimbursement regardless of the level of certainty concerning relative efficacy. However, the results suggest that payers prioritize documented over potential efficacy. Given that updated information on relative efficacy may emerge post-market access, a potential solution to address challenges related to accessibility and affordability in Europe could involve an increased adoption of market entry agreements. These agreements could allow for price adjustments after the presentation of new knowledge regarding relative efficacy, potentially resolving some of the current challenges.

## 1 Introduction

The rising cost of medicines is a significant burden on healthcare systems. Globally, there was a 13% increase in annual expenditure on medicines from 2019 to 2022, independent of COVID-19 (Tichy et al., 2022; Pritchett et al., 2023). This upward trend is primarily attributed to the growth in the cost of new drugs, while increased utilization and prescriptions have had a relatively low impact (Parasrampuria and Murphy, 2022; Pritchett et al., 2023). In the United States, there has been a 20% increase in launch prices for new drugs over the last decade (Rome et al., 2022). To control the growing expenses for pharmaceuticals, European countries are implementing new procurement practices such as reference pricing, public tendering, price discounts, prescription guidelines for physicians, and generic substitution (European Commission, 2022).

The World Health Organization (WHO) recommends implementing health technology assessments (HTAs) to inform reimbursement decisions, a practice adhered to by 40% of all member countries (WHO, 2023). In Norway, the decision on public reimbursement is based on various aspects, including a cost-utility analysis provided by the market authorization (MA) holder and evaluated by the Norwegian Medicinal Products Agency (NOMA). The analysis compares the new drug with the existing treatment alternative and calculates the incremental cost-effectiveness ratio (ICER). The ICER considers both the cost and utility of both the new and the old drug (NICE, 2008). The established relative efficacy, meaning the comparison (direct or indirect) of treatment outcomes between a new drug and standard-of-care for a given indication, provides a more robust estimate of the ICER compared with potential efficacy, meaning single-arm studies, non-adjusted indirect comparisons based on, e.g., response rate or duration of response only.

There has been an increased number of submissions to the European Medicines Agency (EMA) and the U.S. Food and Drug Administration (FDA) based on limited knowledge of relative effect, long-term effect, and side effects due to the use of non-randomized, single-arm studies, surrogate endpoints, and shorter follow-up time (Goring et al., 2019; Del Paggio JC et al., 2021). This trend is partially due to the introduction of expedited approval programs for drugs in situations where comprehensive data cannot be provided and where the benefit of immediate availability outweighs the risk. Limited knowledge of relative efficacy is challenging HTA evaluation and reimbursement decisions (Vreman et al., 2020). To a certain extent, HTA methodologies have adapted; for example, there is an increased use of external control arms. However, this has consequently led to a reduction in the robustness of the HTA evaluation (Burger et al., 2021; Jaksa et al., 2022).

In Norway, HTAs are systematically used at the national level, primarily employing cost-utility analyses as a tool for making informed decisions on whether to introduce new interventions into healthcare services (reimbursement decision). In Norway, the decision-making process for reimbursement considers three prioritization criteria: benefit, resources, and severity. These factors are all incorporated into the reimbursement decision process. A cost-utility analysis provides an assessment of benefit (gain in quality-adjusted life years, QALYs) and resources/incremental costs. Further, the severity of the disease in question is operationalized as an absolute shortfall, measured in QALY loss. However, in cases where the HTA body (NOMA) considers the clinical documentation to be inadequate to establish a robust estimate of relative efficacy, the cost-utility model is not assessed, and hence, no ICER is presented to the payers. In cases where the cost-utility model is not assessed, the priority criteria cannot be evaluated by these tools; hence, a more limited assessment of incremental effect (if applicable) and annual treatment costs (based on confidential net prices) is undertaken. Hence, based on an overview of drugs for which it was possible to present an ICER or not, drugs can be categorized by the robustness of evidence of therapeutic benefit.

This paper summarizes the reimbursement decisions for all new hospital-financed drugs introduced to the Norwegian market between 2021 and 2022. The primary objective is to provide insights into the impact of the level of documentation on the accessibility and affordability of new drugs in the Norwegian healthcare system and to compare accessibility to countries with a similar system for reimbursement. Additional analyses were directed specifically toward oncology drugs, as a substantial proportion of drugs approved by EMA through expedited approval programs, such as conditional approvals are in this therapeutic area (Hwang et al., 2022).

## 2 Methods

All reimbursement decisions, along with corresponding NOMA appraisals, for hospital financed drugs between 1 January 2021, and 31 December 2022, were accessed through www.nyemetoder.no. Decisions that solely considered price per gram, non-drug decisions (e.g., diagnostics), and decisions made without price information were excluded. Only decisions relevant for cost-utility analysis were included (see Supplementary Table S1).

The NOMA appraisals were reviewed to determine whether comparative clinical efficacy was evaluated by NOMA. The drugs were classified into three categories:

1) Drugs with a clinically comparable drug already reimbursed for the given indication (a cost-utility analysis is not considered necessary as the treatment cost of the already reimbursed drug serves as an anchor in a cost-minimization analysis).
2) Drugs presented with an ICER based on a cost-utility model evaluated by NOMA (relative efficacy presented to payers).
3) Drugs presented without an ICER or cost-utility models evaluated by NOMA and without any reimbursed comparable drugs (relative efficacy not presented and no cost-anchor present).

To analyze oncology drugs separately all reimbursement decisions for oncology indications, as defined by NOMA, were reviewed separately.

To analyze the reimbursement decisions and market entry of each category, we compared the proportion of positive approvals and annual treatment costs (standard dosing) per patient using both launch prices and the confidential rebate prices. Information was extracted from publicly available databases on reimbursement decisions (nyemetoder.no) and published HTA reports by NOMA. Confidential rebate prices were accessed through The Norwegian Hospital Procurement Trust. All information about each reimbursement decision was combined and stored at the Norwegian Hospital Procurement Trust. All authors have access to the complete dataset.

Information about the status of reimbursement decisions in England, Sweden, and Denmark at the time of the reimbursement decision in Norway is provided by the Norwegian Hospital Procurement Trust as part of the price information to the payers. The data is publicly available at nyemetoder. no.

Statistical analysis: The differences in cost are based on the average cost in each drug category, while the graphical description is based on z-score normalization.

## 3 Results

**FIGURE 1:**
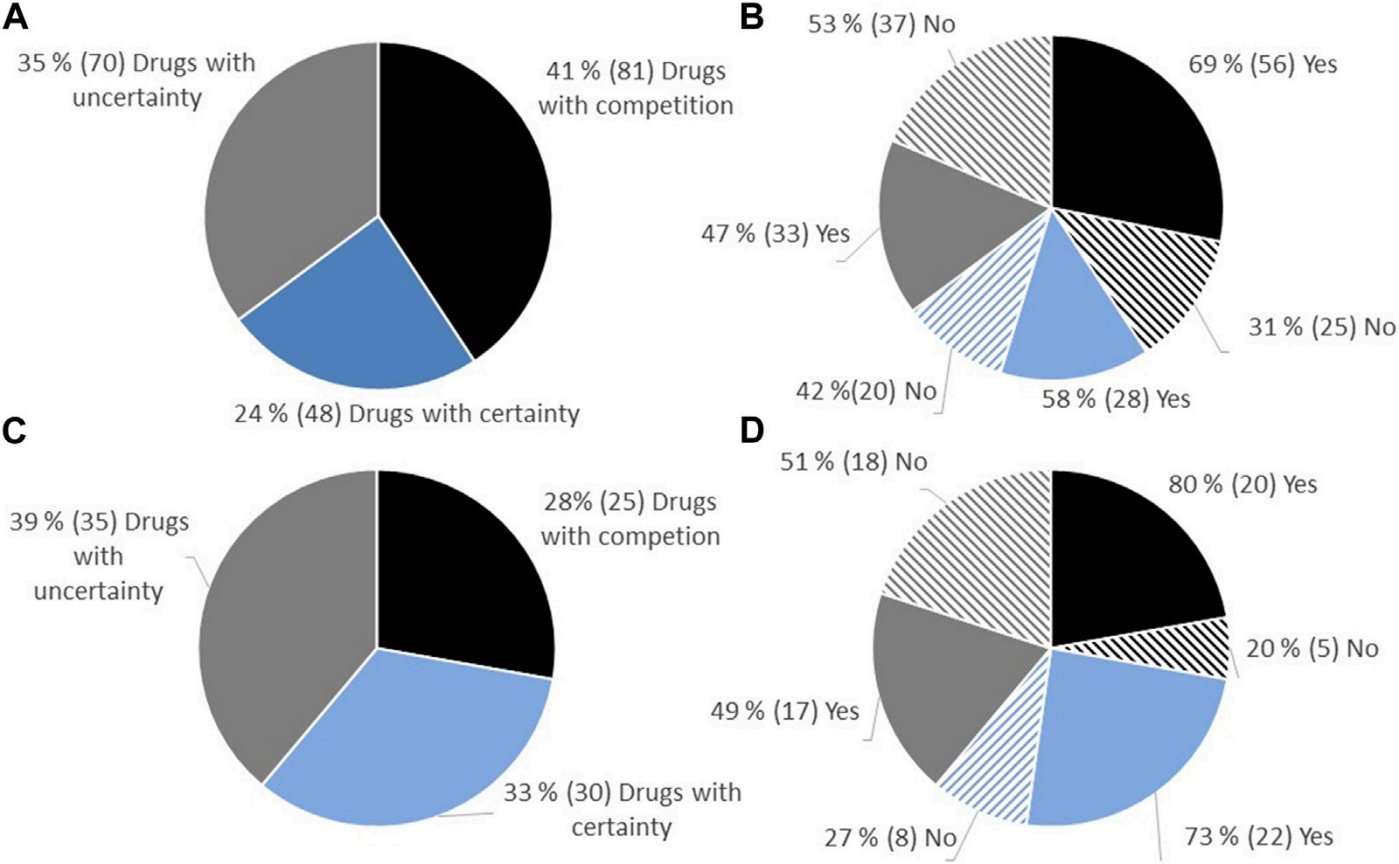
**(A)** Overview of the proportion of approval regarding reimbursement of new hospital-financed drugs in Norway in the period 2021–2022, split into categories depending on the level of uncertainty. **(B)** Proportion of approval in the different categories. **(C)** All decisions on reimbursement of oncology drugs. **(D)** Proportions of approval of oncology drugs depending on category.

Between 2021 and 2022, a total of 238 reimbursement decisions were made for hospital-financed medicinal products in Norway, involving 176 unique medicinal products/indications, as some products had several decisions. Among these decisions, 199 were relevant for cost-efficacy analysis according to the Norwegian reimbursement system (Table 1). Decisions considering only price per gram, non-drug decisions (e.g., diagnostics), and decisions made without price information were excluded. All decisions relevant for cost-utility analysis were included. The drugs were separated into three categories based on the level of documentation regarding therapeutic benefit. Out of the 199 decisions, 41% (81/199) had a clinically comparable drug already reimbursed in Norway, 24% (48/199) had certainty regarding relative efficacy (presented with an ICER), and 35% (70/199) had uncertainty regarding relative efficacy (presented without an ICER) (Figure 1A).

**TABLE 1:** All decisions on reimbursement of hospital financed drugs in Norway in the period 2021–2022. Decisions considering only price per gram, non-drug decisions (e.g., diagnostics), and decisions made without price information were excluded. All decisions relevant for cost-utility analysis were included. CUA: Cost-utility-analysis.

238	Decisions on reimbursement by the regional health authorities in Norway 2021–2022
3	Price per gram
22	Non-drug decisions
14	Decisions without price information
199	Decisions on drugs were CUA were relevant
90	Decisions on oncology drugs were CUA were relevant

Of the 199 decisions in Norway during the period 2021–2022, 45% (90/199) were decisions on oncology drugs. Among these decisions, 28% (25/90) had a comparable drug already reimbursed in Norway, 33% (30/90) had documentation on relative efficacy (presented with an ICER), and 39% (35/90) had limited documentation on relative efficacy (presented without an ICER) (Figure 1B).

### 3.1 Proportion of positive reimbursement decision depending on the robustness regarding evidence of relative efficacy

The proportion of positive reimbursement decisions for each of the three categories was examined. Among drugs entering the market where a clinically comparable drug was already reimbursed, 69% were approved for reimbursement. For drugs with documentation on relative efficacy (presented with an ICER), 58% were approved for reimbursement, while for drugs with limited documentation (no ICER presented), 47% were approved (Figure 1C). Similar proportions of reimbursement approvals were observed for oncology drugs (Figure 1D).

**FIGURE 2:**
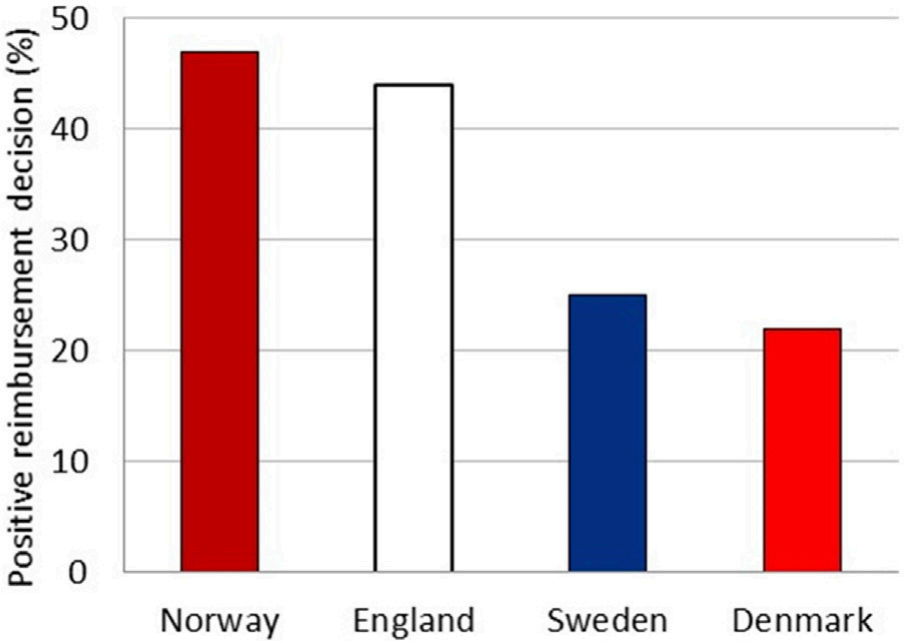
Total percentage of drugs with a positive reimbursement decision in England, Sweden, and Denmark at the time of the decision in Norway.

The reimbursement system in Norway shares similarities with those in Sweden, Denmark, and England. The national launch dates of new drugs are, on average, comparable between the countries (Büssgen and Stargardt, 2022). The documentation requested from the national HTA agencies is similar. However, unlike NOMA, the HTA bodies in Sweden, Denmark, and England evaluate the cost-utility model irrespective of the level of documentation (personal communication, June 2023). To examine the possible impact of the different approach in HTA assessment on access, we compared reimbursement status in Sweden, Denmark, and England at the time of the decision in Norway for all drugs considered by NOMA to have uncertain relative efficacy (Figure 2). England had already approved 44% of these drugs, while Sweden and Denmark had approved 25% and 22%, respectively, compared to a 47% approval rate in Norway. The majority of the drugs not approved in the respective countries were either still under evaluation or not considered for evaluation.

### 3.2 Comparison of annual treatment cost (based on confidential net prices)

**FIGURE 3:**
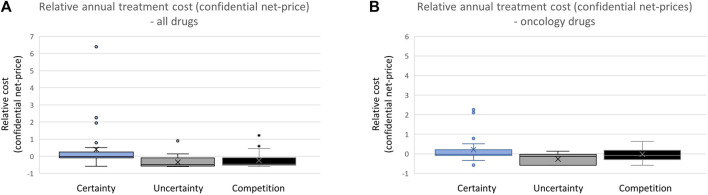
**(A)** Comparing annual treatment cost of all reimbursed drugs in the period 2021–2022. **(B)** Comparing annual treatment cost of all reimbursed oncology drugs.

By comparing the annual treatment costs for the three categories of drugs (Figure 3), we can explore the variation in confidential net prices for reimbursed drugs. Hospital-financed drugs supported by documentation regarding relative efficacy are, on average, accepted for reimbursement with a cost that is 4.4 times higher (confidential net price) than drugs with limited documentation (Figure 3A). Hospital drugs entering the market without clinically comparable drugs already reimbursed and with documentation on relative efficacy are on average accepted with a 4.0 times higher cost (confidential net prices) compared to drugs entering a market where there is already a comparable drug reimbursed.

Focusing specifically on oncology drugs, those with documentation on relative efficacy are on average accepted for reimbursement with a 3.3 times higher cost level (confidential net prices) compared to oncology drugs without such documentation. Oncology drugs entering the market when there is no clinically relevant treatment alternative available are accepted with treatment costs that are on average 1.4 times higher than drugs entering a market with a clinically relevant competing drug already reimbursed (Figure 3B).

### 3.3 Correlation between launch price and confidential net price

**FIGURE 4:**
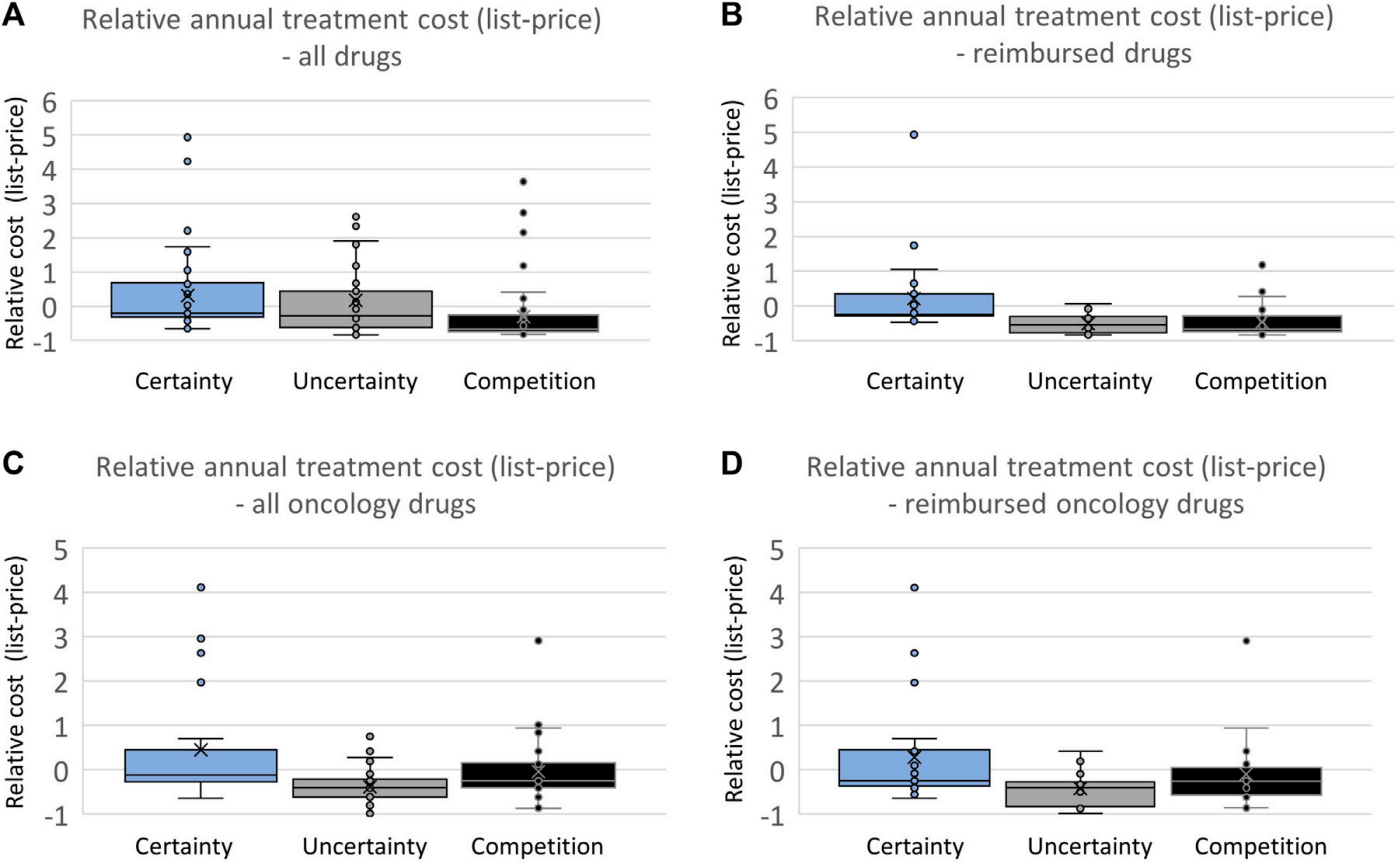
**(A)** Comparing annual treatment cost based on the list price of all drugs. **(B)** Comparing annual treatment cost based on the list price of all reimbursed drugs. **(C)** Comparing annual treatment cost based on the list price of all oncology drugs. **(D)** Comparing annual treatment cost based on the list price of all reimbursed oncology drugs.

To investigate whether the difference in the certainty of estimated relative efficacy is reflected in the pricing strategies of pharmaceutical companies, an analysis of the cost difference was conducted based on the list-price of all drugs and reimbursed drugs separately (Figure 4). The comparisons of list prices for the three categories were performed by considering annual treatment costs per patient. There was a high variation in list prices in all categories, and no trend towards differences in list prices of drugs based on the level of documentation was observed (Figure 4A). However, drugs with a comparable drug already on the market had a significantly lower list price (2.2 times lower on average) compared to drugs without such competition. When considering only reimbursed drugs (Figure 4B), the difference reemerged. Drugs with robust documentation on relative efficacy (presented with an ICER) were on average 3 times more expensive than drugs with less robust documentation (without an ICER) and 2.8 times more expensive than drugs with clinically comparable competition.

When examining oncology drugs separately, both drugs with uncertainty and drugs with competition had an average lower list price (1.7 and 1.3, respectively) compared with drugs with certainty regarding relative efficacy (Figures 4C, D). The same pattern can be seen for reimbursed oncology drugs, with drugs with certainty regarding relative efficacy having an average list price 1.7 and 1.3 times higher than drugs with uncertainty regarding relative efficacy or competition already on the market, respectively.

## 4 Discussion

The Norwegian reimbursement system is based on cost-utility analyses, providing an assessment of utility (gain in quality-adjusted life years, QALYs) and resources/incremental costs. In cases where the HTA agency (NOMA) deems the clinical documentation inadequate for establishing a robust estimate of relative efficacy, the cost-utility model remains unassessed, leading to the absence of an ICER. The results presented here indicate that a significant number (35 %) of reimbursement decisions are based on limited documentation regarding the relative efficacy drugs. A similar pattern is observed when analyzing oncology drugs separately. This aligns with the development in clinical trial methodologies, characterized by the utilization of surrogate endpoints, shorter follow-up periods, and single-arm trials, all of which have introduced increased uncertainties in the HTA process (Goring et al., 2019; Grimm et al., 2019; Del Paggio et al., 2021; Trapani et al., 2022; Merino et al., 2023).

Uncertainty about efficacy may result in delays in the pricing and reimbursement process, as therapeutic value and the quality of evidence are decisive factors for reimbursement (Malinowski et al., 2018; Galeone et al., 2021; Jommi et al., 2021; Siegmeier and Büssgen, 2022; EFPIA, 2023). This is also seen in Norway, where a lower proportion of reimbursement approvals is observed for drugs with limited documentation available. Similar patterns emerge when examining oncology drugs separately. These findings underscores that the level of documentation of relative efficacy and the presence of comparable drugs already reimbursed influence the probability of reimbursement in Norway.

Approximately half of all drugs approved by the EMA demonstrate meaningful clinical benefit according to the European Society for Medical Oncology (ESMO) Magnitude of Clinical Benefit Scale (ESMO-MCBS) grades (Booth and Del Paggio, 2017; Vivot et al., 2017; Tibau et al., 2018). However, several studies show no consistent relation between assumed clinical benefit and cost (Vivot et al., 2017; Mailankody and Prasad, 2015; Salas-Vega et al., 2020; Saluja et al., 2018; Vokinger et al., 2020). In Italy, examining confidential net prices revealed a correlation between the annual cost of drugs and therapeutic benefit (Jommi et al., 2021). This finding is consistent with our results from Norway, where a lack of evidence of added therapeutic benefit correlates with lower drug costs (confidential net prices). These results emphasize the importance of documented clinical benefit when considering reimbursement of new drugs, and documentation of relative efficacy justifies higher cost levels when drugs enter the Norwegian market.

When considering list prices for all drugs, no differences were observed between drugs. However, when looking only at drugs accepted for reimbursement, the cost difference reemerged, indicating that some companies have a pricing strategy reflecting the current level of documentation regarding relative efficacy. In Europe, there is an increasing utilization of managed entry agreements to address challenges associated with escalating drug costs and heightened uncertainty regarding clinical benefits (Ciulla et al., 2023). Interestingly, competition from on-patent clinically comparable drugs reduced both the list price and the confidential net price of new drugs. This effect was observed even when considering only oncology drugs. In terms of confidential net price, this outcome may reflect the utilization of tendering processes for on-patent clinically comparable drugs in Norway. If a new drug within a treatment group wins the tender, it can acquire a significant market share, leading to 70%–100% of all new patients starting treatment with the new drug.

All oncology drugs receiving accelerated approval by the FDA before November 2018 have been converted to traditional approval through supplementary confirmatory studies (Beaver et al., 2018; Subbiah et al., 2022). EMA’s human medicines committee (CHMP) recently recommended not renewing the conditional marketing authorisation for Blenrep (belantamab mafodotin), a medicine used to treat multiple myeloma. At the time of the initial authorisation, no comparative data for Blenrep were available. The recent recommendation follows a review of available data by the CHMP as part of the renewal of Blenrep’s marketing authorisation. In its review, the CHMP considered that results from a new study did not confirm the effectiveness of Blenrep as agreed when conditional marketing authorisation was granted (EMA, 2023). A reevaluation of cost-efficacy analyses reveals a high degree of variation between pre- and post-market entry (Guggenbickler et al., 2022), highlighting the disparity between the estimated patient benefit at the time of market entry and the perceived patient benefit in clinical practice. This aligns with observational studies examining survival data, indicating improvement in survival for certain cancer indications, while demonstrating limited or no effect in others (Neyt et al., 2023). In conclusion, early market entry heightens the risk of introducing inefficient drugs, into the clinical setting without a comprehensive follow-up plan aimed at closing knowledge gaps and with option to reassess reimbursement decisions. Monitoring post-marketing efficacy should be conducted with the same level of rigor as post-marketing safety. Extensive long-term analyses have revealed that approximately 70 % of FDA approved orphan drugs undergo safety-related labeling changes, although severe safety events are rare (Fan et al., 2022). The implementation of post-marketing surveillance serves the dual purpose of ensuring early access to treatments while concurrently prioritizing patient safety.

The discrepancy between perceived and documented value can be addressed through the implementation of managed entry agreements, as evidenced by the increasing adoption of such agreements (Jommi et al., 2020; Efthymiadou and Kanavos, 2022). The complexity associated with managed entry agreement implementation remains a challenge and contributes to extended time frames for the final reimbursement decision (Kang et al., 2020; Eichler et al., 2021; Fens et al., 2021). To optimize the utilization of managed entry agreements, it is essential to incorporate them into the pricing strategies of pharmaceutical companies. A mutually agreed-upon strategy for assessing the clinical benefit of new drugs is crucial for ensuring patient access (Pignatti et al., 2022; Xoxi et al., 2022). Both the pharmaceutical industry and regulatory entities recognize that, in some situations where a randomized study is not feasible, real-world data can offer a valuable comparison to quantify relative efficacy. Nonetheless, moving forward, the development of clear guidelines will be necessary to guide the use of real-world data in such contexts (Burger et al., 2021).

Reimbursement agencies are mainly concerned with proven health gain when procuring new drugs. However, incentives for innovation are important for the development of new drugs, as emphasized by the EU pharmaceutical strategy (European Commission, 2020). This is supported by providing the possibility of early market entry, but for this to be successful, it must also lead to reimbursement. To achieve this aim, the pricing and market strategy should reflect the level of documentation at market entry. However, often more information regarding relative efficacy comes after market entry. One strategy can be the use of managed entry agreements that allow for a reduced price level at the time of reimbursement and potential price increase over time if new documentation on relative efficacy is provided.

## Data Availability

The datasets presented in this article are not readily available because the confidential net-prices cannot be shared. Requests to access the datasets should be directed to www.sykehusinnkjop.no.
