# Supplementary material for: The impact of level of documentation on the accessibility and affordability of new drugs in Norway
Source: Front Pharmacol. 2024 Feb 14;15:1338541. doi: 10.3389/fphar.2024.1338541 (PMC10899517; doi:10.3389/fphar.2024.1338541)
Supplement: Supplementary file 1 [file Table1.docx]

Supplementary Material

# Supplementary Data

Supplementary Material should be uploaded separately on submission. Please include any supplementary data, figures and/or tables.

Supplementary material is not typeset so please ensure that all information is clearly presented, the appropriate caption is included in the file and not in the manuscript, and that the style conforms to the rest of the article.

# Supplementary Figures and Tables

For more information on Supplementary Material and for details on the different file types accepted, please see [here](https://www.frontiersin.org/guidelines/author-guidelines#supplementary-material).

**Supplementary:**

Table 1: Overview of included drugs

| Identification number | Drug name |
| --- | --- |
| [ID2017_054](https://nyemetoder.no/metoder/abirateron-indikasjon-iii) | Abirateron (Zytiga) |
| [ID2021_138](https://nyemetoder.no/metoder/abirateron-indikasjon-iv) | Abirateron (Zytiga) |
| [ID2022_032](https://nyemetoder.no/metoder/abirateron-indikasjon-v) | Abirateron (Zytiga) |
| [ID2021_053](https://nyemetoder.no/metoder/abrocitinib-cibinqo) | Abrocitinib (Cibinqo) |
| [ID2020_037](https://nyemetoder.no/metoder/akalabrutinib-calquence) | Acalabrutinib (Calquence) |
| [ID2021_086](https://nyemetoder.no/metoder/akalabrutinib-calquence-indikasjon-ii) | Acalabrutinib (Calquence) |
| [ID2021_087](https://nyemetoder.no/metoder/akalabrutinib-calquence-indikasjon-iii) | Acalabrutinib (Calquence) |
| [ID2019_070](https://nyemetoder.no/metoder/alpelisib-piqray) | Alpelisib (Piqray) |
| [ID2019_138](https://nyemetoder.no/metoder/aminolevulinsyre-ameluz) | Aminolevulinic acid (Ameluz) |
| [ID2019_144](https://nyemetoder.no/metoder/anakinra-kineret-indikasjon-ii) | Anakinra (Kineret) |
| [ID2019_113](https://nyemetoder.no/metoder/apalutamid-erleada-indikasjon-ii) | Apalutamide (Erleada) |
| [ID2015_002](https://nyemetoder.no/metoder/apremilast-otezla) | Apremilast (Otezla) |
| [ID2019_062](https://nyemetoder.no/metoder/ataluren-translarna-indikasjon-ii) | Ataluren (Translarna) |
| [ID2019_044](https://nyemetoder.no/metoder/atezolizumab-tecentriq-indikasjon-vii) | Atezolizumab (Tecentriq) |
| [ID2020_006](https://nyemetoder.no/metoder/atezolizumab-tecentriq-indikasjon-ix) | Atezolizumab (Tecentriq) |
| [ID2020_030](https://nyemetoder.no/metoder/atezolizumab-tecentriq-indikasjon-x) | Atezolizumab (Tecentriq) |
| [ID2021_034](https://nyemetoder.no/metoder/atezolizumab-tecentriq-indikasjon-xviii) | Atezolizumab (Tecentriq) |
| [ID2020_043](https://nyemetoder.no/metoder/avatrombopag-doptelet-indikasjon-ii) | Avatrombopag (Doptelet) |
| [ID2018_131](https://nyemetoder.no/metoder/avatrombopaq-doptelet) | Avatrombopaq (Doptelet) |
| [ID2020_083](https://nyemetoder.no/metoder/avelumab-bavencio-indikasjon-iii) | Avelumab (Bavencio) |
| [ID2019_143](https://nyemetoder.no/metoder/axicabtagene-ciloleucel-yescarta) | Axicabtagene ciloleucel (Yescarta) |
| [ID2020_055](https://nyemetoder.no/metoder/belantamabmafodotin-blenrep) | Belantamab mafodotin (Blenrep) |
| [ID2020_088](https://nyemetoder.no/metoder/belimumab-benlysta) | Belimumab (Benlysta) |
| [ID2021_048](https://nyemetoder.no/metoder/berotralstat-orladeyo) | Berotralstat (Orladeyo) |
| [ID2021_013](https://nyemetoder.no/metoder/bimekizumab-bimzelx) | Bimekizumab (Bimzelx) |
| [ID2019_118](https://nyemetoder.no/metoder/blinatumomab-blincyto-indikasjon-iii) | Blinatumomab (Blincyto) |
| [ID2019_114](https://nyemetoder.no/metoder/brentuksimabvedotin-adcetris-indikasjon-v) | Brentuximab vedotin (Adcetris) |
| [ID2019_092](https://nyemetoder.no/metoder/brigatinib-alunbrig-indikasjon-ii) | Brigatinib (Alunbrig) |
| [ID2021_132](https://nyemetoder.no/metoder/brolucizumab-beovu-indikasjon-ii) | Brolucizumab (Beovu) |
| [ID2021_103](https://nyemetoder.no/metoder/budesonid-jorveza-indikasjon-ii) | Budesonide (Jorveza) |
| [ID2019_050](https://nyemetoder.no/metoder/buprenorfin-subutex-depotinjeksjonsveske) | Buprenorphine (Subutex) |
| [ID2017_076](https://nyemetoder.no/metoder/buprenorfinnalokson-zubsolv) | Buprenorphine/ naloxone (Zubsolv) |
| [ID2020_097](https://nyemetoder.no/metoder/buprenorfinimplantat-sixmo) | Buprenorphine (Sixmo) |
| [ID2018_021](https://nyemetoder.no/metoder/burosumab-crysvita) | Burosumab (Crysvita) |
| [ID2020_025](https://nyemetoder.no/metoder/canakinumab-ilaris-indikasjon-ii) | Canakinumab (Ilaris) |
| [ID2018_081](https://nyemetoder.no/metoder/cannabidiol-epidyolex) | Cannabidiol (Epidyolex) |
| [ID2020_094](https://nyemetoder.no/metoder/cannabidiol-tetrahydrocannabinol-sativex) | Cannabidiol / tetrahydrocannabinol (Sativex) |
| [ID2019_125](https://nyemetoder.no/metoder/cefiderokol-fetcroja) | Cefiderocol (Fetcroja) |
| [ID2021_007](https://nyemetoder.no/metoder/cemiplimab-libtayo-indikasjon-ii) | Cemiplimab (Libtayo) |
| [ID2021_008](https://nyemetoder.no/metoder/cemiplimab-libtayo-indikasjon-iii) | Cemiplimab (Libtayo) |
| [ID2017_033](https://nyemetoder.no/metoder/cerliponase-alfa-brineura) | Cerliponase alfa (Brineura) |
| [ID2018_068](https://nyemetoder.no/metoder/certolizumab-pegol-cimzia) | Certolizumab pegol (Cimzia) |
| [ID2019_078](https://nyemetoder.no/metoder/daratumumab-darzalex-bortezomib-talidomid-og-deksametason) | Daratumumab (Darzalex) |
| [ID2019_105](https://nyemetoder.no/metoder/darolutamid-nubeqa) | Darolutamide (Nubeqa) |
| [ID2021_099](https://nyemetoder.no/metoder/diroksimelfumarat-vumerity) | Diroximel fumarate (Vumerity) |
| [ID2019_068](https://nyemetoder.no/metoder/dupilumab-dupixent-indikasjon-iv) | Dupilumab (Dupixent) |
| [ID2020_036](https://nyemetoder.no/metoder/dupilumab-dupixent-indikasjon-v) | Dupilumab (Dupixent) |
| [ID2021_121](https://nyemetoder.no/metoder/dupilumab-dupixent-indikasjon-vi) | Dupilumab (Dupixent) |
| ID2017_020 | Eculizumab (Soliris) |
| ID2019_043 | Eculizumab (Soliris) |
| [ID2019_061](https://www.nyemetoder.no/metoder/ekulizumab-soliris-indikasjon-iii) | Eculizumab (Soliris) |
| ID2020_003 | Eculizumab (Soliris) |
| [ID2020_029](https://nyemetoder.no/metoder/elexakaftor-tezakaftor-ivakaftor-kaftrio) | Elexacaftor/tezacaftor/ ivacaftor (Kaftrio) |
| [ID2020_018](https://nyemetoder.no/metoder/elosulfase-alfa-vimizim) | Elosulfase alfa (Vimizim) |
| [ID2018_040](https://nyemetoder.no/metoder/elotuzumab-empliciti) | Elotuzumab (Empliciti) |
| [ID2018_066](https://nyemetoder.no/metoder/emicizumab-hemlibra-indikasjon-ii) | Emicizumab (Hemlibra) |
| [ID2019_083](https://nyemetoder.no/metoder/encorafenib-braftovi-og-cetuximab-erbitux) | Encorafenib (Braftovi) |
| [ID2019_115](https://nyemetoder.no/metoder/entrektinib-rozlytrek) | Entrectinib (Rozlytrek) |
| [ID2019_119](https://nyemetoder.no/metoder/entrektinib-rozlytrek-indikasjon-ii) | Entrectinib (Rozlytrek) |
| [ID2019_104](https://nyemetoder.no/metoder/enzalutamid-xtandi-indikasjon-iv) | Enzalutamide (Xtandi) |
| [ID2021_003](https://nyemetoder.no/metoder/enzalutamid-xtandi-indikasjon-iii-revurdering) | Enzalutamide (Xtandi) |
| [ID2016_003](https://nyemetoder.no/metoder/eribulin-halaven-indikasjon-ii) | Eribulin (Halaven) |
| [ID2019_116](https://nyemetoder.no/metoder/esketamin-spravato) | Esketamine (Spravato) |
| [ID2016_054](https://nyemetoder.no/metoder/etelcalcetid-velcalcetid-parsabiv) | Etelcalcetide (velcalcetid/Parsabiv) |
| [ID2020_095](https://nyemetoder.no/metoder/fampridin-fampyra) | Fampridine (Fampyra) |
| [ID2022_046](https://nyemetoder.no/metoder/faricimab-vabysmo-indikasjon-ii) | Faricimab (Vabysmo) |
| [ID2022_045](https://nyemetoder.no/metoder/faricimab-vabysmo) | Faricimab (Vabysmo) |
| [ID2021_140](https://nyemetoder.no/metoder/fedratinib-inrebic-indikasjon-ii) | Fedratinib (Inrebic) |
| [ID2021_014](https://nyemetoder.no/metoder/filgotinib-jyseleca-indikasjon-ii) | Filgotinib (Jyseleca) |
| [ID2014_025](https://nyemetoder.no/metoder/fingolimod-) | Fingolimod (Gilenya) |
| [ID2019_041](https://nyemetoder.no/metoder/fostamatinib-tavlesse) | Fostamatinib (Tavlesse) |
| [ID2020_073](https://nyemetoder.no/metoder/fostemsavir-rukobia) | Fostemsavir (Rukobia) |
| [ID2019_095](https://nyemetoder.no/metoder/gilteritinib-xospata) | Gilteritinib (Xospata) |
| [ID2020_014](https://nyemetoder.no/metoder/givosiran-givlaari) | Givosiran (Givlaari) |
| [ID2021_044](https://nyemetoder.no/metoder/glukarpidase-voraxaze) | Glucarpidase (Voraxaze) |
| [ID2017_065](https://nyemetoder.no/metoder/guselkumab-tremfya) | Guselkumab (Tremfya) |
| [ID2019_140](https://nyemetoder.no/metoder/guselkumab-tremfya-indikasjon-ii) | Guselkumab (Tremfya) |
| [ID2019_020](https://nyemetoder.no/metoder/human-c1-esterasehemmer-berinert) | Human C1 Esterase Inhibitor (Berinert) |
| [ID2021_072](https://nyemetoder.no/metoder/hydrokortison-efmody) | Hydrocortisone (Efmody) |
| [ID2022_057](https://nyemetoder.no/metoder/hydroksykarbamid-siklos-indikasjon-ii) | Hydroxycarbamide (Siklos) |
| [ID2019_028](https://nyemetoder.no/metoder/ibalizumab-trogarzo) | Ibalizumab (Trogarzo) |
| [ID2015_010](https://nyemetoder.no/metoder/ibrutinib-imbruvica-indikasjon-iii) | Ibrutinib (Imbruvica) |
| [ID2019_016](https://nyemetoder.no/metoder/ibrutinib-imbruvica-indikasjon-vi) | Ibrutinib (Imbruvica) |
| [ID2020_033](https://nyemetoder.no/metoder/ibrutinib-imbruvica-indikasjon-viii) | Ibrutinib (Imbruvica) |
| [ID2016_002](https://nyemetoder.no/metoder/ibrutinib-imbruvica-indikasjon-iv) | Ibrutinib (Imbruvica) |
| [ID2018_059](https://nyemetoder.no/metoder/ixazomib-ninlaro) | Ixazomib (Ninlaro) |
| [ID2019_130](https://nyemetoder.no/metoder/iksekizumab-taltz-indikasjon-iv) | Ixekizumab (Taltz) |
| [ID2019_102](https://nyemetoder.no/metoder/imlifidase-idefirix) | Imlifidase (Idefirix) |
| [ID2020_056](https://nyemetoder.no/metoder/ipilimumab-yervoy-nivolumab-opdivo-indikasjon-v) | Ipilimumab (Yervoy) / nivolumab (Opdivo) |
| [ID2019_137](https://nyemetoder.no/metoder/isatuksimab-sarclisa) | Isatuximab (Sarclisa) |
| [ID2020_012](https://nyemetoder.no/metoder/kabotegravir-vocabria) | Cabotegravir (Vocabria) |
| [ID2020_105](https://nyemetoder.no/metoder/kabozantinib-cabometyx-nivolumab-opdivo) | Cabozantinib (Cabometyx) /nivolumab (Opdivo) |
| [ID2015_005](https://nyemetoder.no/metoder/karfilzomib-kyprolis) | Carfilzomib (Kyprolis) |
| [ID2020_027](https://nyemetoder.no/metoder/katridekakog-novothirteen) | Catridecacog (NovoThirteen) |
| [ID2018_096](https://nyemetoder.no/metoder/klormetin-ledaga) | Chlormethine (Ledaga) |
| [ID2018_093](https://nyemetoder.no/metoder/lanadelumab-takhzyro) | Lanadelumab (Takhzyro) |
| [ID2020_075](https://nyemetoder.no/metoder/landiololhydroklorid-raploc) | Landiolol hydrochloride (Raploc) |
| [ID2019_029](https://nyemetoder.no/metoder/larotrectinib-vitrakvi) | Larotrectinib (Vitrakvi) |
| [ID2017_049](https://nyemetoder.no/metoder/lenalidomid-indikasjon-ii) | Lenalidomide (Revlimid) |
| [ID2017_050](https://nyemetoder.no/metoder/lenalidomid-indikasjon-iii) | Lenalidomide (Revlimid) |
| [ID2017_051](https://nyemetoder.no/metoder/lenalidomid-indikasjon-iv) | Lenalidomide(Revlimid) |
| [ID2018_109](https://nyemetoder.no/metoder/lenalidomid-indikasjon-v) | Lenalidomide (Revlimid) |
| [ID2019_054](https://nyemetoder.no/metoder/lenalidomid-indikasjon-vi) | Lenalidomide (Revlimid) |
| [ID2021_079](https://nyemetoder.no/metoder/lenvatinib-kisplyx-pembrolizumab-keytruda) | Lenvatinib (Kisplyx) / pembrolizumab (Keytruda) |
| [ID2018_115](https://nyemetoder.no/metoder/lomitapid-lojuxta) | Lomitapide (Lojuxta) |
| [ID2021_066](https://nyemetoder.no/metoder/lorlatinib-lorviqua-indikasjon-ii) | Lorlatinib (Lorviqua) |
| [ID2020_070](https://nyemetoder.no/metoder/mannitol-bronchitol) | Mannitol (Bronchitol) |
| [ID2020_047](https://nyemetoder.no/metoder/meksiletin-namuscla) | Mexiletine hcl (Namuscla) |
| [ID2020_039](https://nyemetoder.no/metoder/metreleptin-myalepta) | Metreleptin (Myalepta) |
| [ID2018_032](https://nyemetoder.no/metoder/mogamulizumab-poteligeo) | Mogamulizumab (Poteligeo) |
| [ID2021_019](https://nyemetoder.no/metoder/natalizumab-tysabri-indikasjon-iv) | Natalizumab (Tysabri) |
| [ID2019_136](https://nyemetoder.no/metoder/nintedanib-ofev-indikasjon-iii) | Nintedanib (Ofev) |
| [ID2020_034](https://nyemetoder.no/metoder/niraparib-zejula-indikasjon-iv) | Niraparib (Zejula) |
| [ID2021_051](https://nyemetoder.no/metoder/niraparib-zejula-indikasjon-ii-revurdering) | Niraparib (Zejula) |
| [ID2020_050](https://nyemetoder.no/metoder/niraparib-zejula-indikasjon-v) | Niraparib (Zejula) |
| [ID2020_026](https://nyemetoder.no/metoder/nivolumab-opdivo-indikasjon-xiii) | Nivolumab (Opdivo) |
| [ID2021_040](https://nyemetoder.no/metoder/nivolumab-opdivo-indikasjon-xiv) | Nivolumab (Opdivo) |
| [ID2019_134](https://nyemetoder.no/metoder/normalt-humant-immunglobulin-cutaquig) | Human Normal Immunoglobulin (Cutaquig) |
| [ID2020_066](https://nyemetoder.no/metoder/ofatumumab-kesimpta-indikasjon-iv) | Ofatumumab (Kesimpta) |
| [ID2020_009](https://nyemetoder.no/metoder/olaparib-lynparza-indikasjon-vii) | Olaparib (Lynparza) |
| [ID2019_066](https://nyemetoder.no/metoder/osilodrostat-isturisa-) | Osilodrostat (Isturisa) |
| [ID2018_005](https://nyemetoder.no/metoder/osimertinib-tagrisso-indikasjon-ii) | Osimertinib (Tagrisso) |
| [ID2018_057](https://nyemetoder.no/metoder/osimertinib-tagrisso) | Osimertinib (Tagrisso) |
| [ID2020_106](https://nyemetoder.no/metoder/osimertinib-tagrisso-indikasjon-iii) | Osimertinib (Tagrisso) |
| [ID2021_042](https://nyemetoder.no/metoder/ozanimod-zeposia-indikasjon-ii) | Ozanimod (Zeposia) |
| [ID2021_049](https://nyemetoder.no/metoder/patiromer-veltassa) | Patiromer (Veltassa) |
| [ID2021_054](https://nyemetoder.no/metoder/pegcetacoplan-aspaveli) | Pegcetacoplan (Aspaveli) |
| [ID2018_100](https://nyemetoder.no/metoder/pegvaliase-palynziq) | Pegvaliase (Palynziq) |
| [ID2020_078](https://nyemetoder.no/metoder/pembrolizumab-keytruda-indikasjon-xvi) | Pembrolizumab (Keytruda) |
| [ID2020_082](https://nyemetoder.no/metoder/pembrolizumab-keytruda-indikasjon-xvii) | Pembrolizumab (Keytruda) |
| [ID2021_030](https://www.nyemetoder.no/metoder/pembrolizumab-keytruda-indikasjon-xviii) | Pembrolizumab (Keytruda) |
| [ID2021_039](https://nyemetoder.no/metoder/pembrolizumab-keytruda-indikasjon-xix) | Pembrolizumab (Keytruda) |
| [ID2021_080](https://nyemetoder.no/metoder/lenvatinib-lenvima-pembrolizumab-keytruda) | Lenvatinib (Lenvima) / pembrolizumab (Keytruda) |
| [ID2020_059](https://nyemetoder.no/metoder/pemigatinib-pemazyre) | Pemigatinib (Pemazyre) |
| [ID2020_064](https://nyemetoder.no/metoder/pertuzumab-trastuzumab-phesgo) | Pertuzumab, trastuzumab (Phesgo) |
| [ID2019_035](https://nyemetoder.no/metoder/polatuzumabvedotin-polivy-) | Polatuzumab vedotin (Polivy) |
| [ID2020_089](https://nyemetoder.no/metoder/ponesimod-ponvory) | Ponesimod (Ponvory) |
| [ID2020_108](https://nyemetoder.no/metoder/pralsetinib-gavreto) | Pralsetinib (Gavreto) |
| [ID2021_095](https://nyemetoder.no/metoder/risankizumab-skyrizi-indikasjon-ii) | Risankizumab (Skyrizi) |
| [ID2021_088](https://nyemetoder.no/metoder/risdiplam-evrysdi-indikasjon-ii) | Risdiplam (Evrysdi) |
| [ID2020_107](https://nyemetoder.no/metoder/roksadustat-evrenzo) | Roxadustat (Evrenzo) |
| [ID2020_013](https://nyemetoder.no/metoder/satralizumab-enspryng) | Satralizumab (Enspryng) |
| [ID2020_076](https://nyemetoder.no/metoder/selperkatinib-retsevmo) | Selpercatinib (Retsevmo) |
| [ID2020_077](https://nyemetoder.no/metoder/selperkatinib-retsevmo-indikasjon-ii) | Selpercatinib (Retsevmo) |
| [ID2020_099](https://nyemetoder.no/metoder/selperkatinib-retsevmo-indikasjon-iii) | Selpercatinib (Retsevmo) |
| [ID2019_040](https://nyemetoder.no/metoder/siponimod-mayzent) | Siponimod (Mayzent) |
| [ID2021_109](https://nyemetoder.no/metoder/sofosbuvir-velpatasvir-og-voxilaprevir-vosevi-i-fast-kombinasjon-indikasjon-ii) | Sofosbuvir, velpatasvir, voxilaprevi (Vosevi) |
| [ID2021_035](https://nyemetoder.no/metoder/sonidegib-odomzo) | Sonidegib (Odomzo) |
| [ID2020_079](https://nyemetoder.no/metoder/streptozocin-zanosar) | Streptozocin (Zanosar) |
| [ID2019_038](https://nyemetoder.no/metoder/tafamidis-vyndaqel-indikasjon-ii) | Tafamidis (Vyndaqel) |
| [ID2018_129](https://nyemetoder.no/metoder/talazoparib-talzenna) | Talazoparib (Talzenna) |
| [ID2021_110](https://nyemetoder.no/metoder/teriflunomid-aubagio-indikasjon-ii) | Teriflunomide (Aubagio) |
| [ID2018_112](https://nyemetoder.no/metoder/tezakaftorivakaftor-symkevi) | Tezacaftor, ivacaftor (Symkevi) |
| [ID2017_102](https://nyemetoder.no/metoder/tildrakizumab-ilumetri) | Tildrakizumab (Ilumetri) |
| [ID2019_141](https://nyemetoder.no/metoder/tisagenlecleucel-kymriah-indikasjon-ii) | Tisagenlecleucel (Kymriah) |
| [ID2020_098](https://nyemetoder.no/metoder/tocilizumab-roactemra-ny-vurdering) | Tocilizumab (RoActemra) |
| [ID2017_108](https://nyemetoder.no/metoder/tofacitinib-xeljanz-indikasjon-ii) | Tofacitinib (Xeljanz) |
| [ID2018_029](https://nyemetoder.no/metoder/tofacitinib-xeljanz-indikasjon-iii) | Tofacitinib (Xeljanz) |
| [ID2021_052](https://nyemetoder.no/metoder/tofacitinib-xeljanz-indikasjon-iv) | Tofacitinib (Xeljanz) |
| [ID2021_093](https://nyemetoder.no/metoder/tofacitinib-xeljanz-indikasjon-v) | Tofacitinib (Xeljanz) |
| [ID2021_005](https://nyemetoder.no/metoder/tralokinumab-adtralza) | Tralokinumab (Adtralza) |
| [ID2021_032](https://nyemetoder.no/metoder/trastuzumab-indikasjon-iii) | Trastuzumab |
| [ID2021_006](https://nyemetoder.no/metoder/trastuzumabderukstekan-enhertu) | Trastuzumab deruxteca (Enhertu) |
| [ID2022_041](https://nyemetoder.no/metoder/trastuzumabderukstekan-enhertu-indikasjon-iii) | Trastuzumab deruxteca (Enhertu) |
| [ID2018_106](https://nyemetoder.no/metoder/trientindihydroklorid-cufence) | Trientine dihydrochloride (Cufence) |
| [ID2019_013](https://nyemetoder.no/metoder/trifluridine-tipiracil-lonsurf-indikasjon-ii) | Trifluridine, tipiracil (Lonsurf) |
| [ID2020_067](https://nyemetoder.no/metoder/tucatinib-tukysa-) | Tucatinib (Tukysa) |
| [ID2020_080](https://nyemetoder.no/metoder/upadacitinib-rinvoq-indikasjon-ii) | Upadacitinib (Rinvoq) |
| [ID2020_081](https://nyemetoder.no/metoder/upadacitinib-rinvoq-indikasjon-iii) | Upadacitinib (Rinvoq) |
| [ID2021_085](https://nyemetoder.no/metoder/upadacitinib-rinvoq-indikasjon-iv-) | Upadacitinib (Rinvoq) |
| [ID2022_044](https://nyemetoder.no/metoder/upadacitinib-rinvoq-indikasjon-v) | Upadacitinib (Rinvoq) |
| [ID2019_112](https://nyemetoder.no/metoder/ustekinumab-stelara-indikasjon-iii) | Ustekinumab (Stelara) |
| [ID2021_076](https://nyemetoder.no/metoder/velmanase-alfa-lamzede) | Velmanase alfa (Lamzede) |
| [ID2019_100](https://nyemetoder.no/metoder/venetoklaks-venclyxto-indikasjon-v) | Venetoclax (Venclyxto) |
| [ID2016_057](https://nyemetoder.no/metoder/voretigene-neparvovec-luxturna) | Voretigene neparvovec (Luxturna) |
| [ID2021_010](https://nyemetoder.no/metoder/zanubrutinib-brukinsa) | Zanubrutinib (Brukinsa) |

Table 1: An overview over included drugs (duplicated decisions removed). The ID number **refers** to the unique ID used for reimbursement request for a specific indication.

**
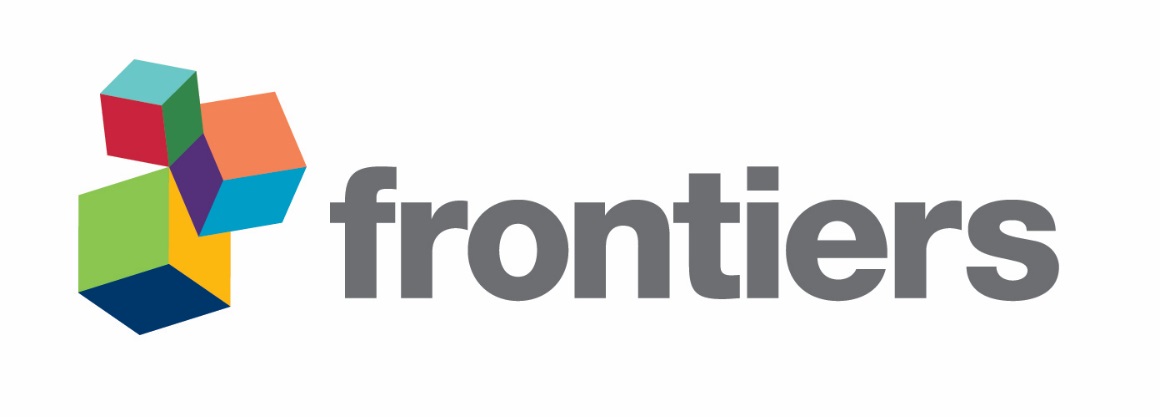
**
